# Risk factors for positive depression screening across a shipboard deployment cycle

**DOI:** 10.1192/bjo.2019.70

**Published:** 2019-09-20

**Authors:** Alice E. Arcury-Quandt, Judith Harbertson, Lauretta Ziajko, Braden R. Hale

**Affiliations:** Project Coordinator, Statistics & Epidemiology Branch, Walter Reed Army Institute of Research; and Laulima Government Solutions, USA; Senior Research Epidemiologist, Leidos Inc; and Department of Defense HIV/AIDS Prevention Program, Defense Health Agency, USA; Associate Program Director Psychiatry Residency, Department of Mental Health Services, Naval Medical Center San Diego, USA; Deputy Division Chief, Department of Defense HIV/AIDS Prevention Program, Defense Health Agency; and Division of Infectious Diseases & Global Public Health, Department of Medicine, University of California San Diego, USA

**Keywords:** Military psychiatry, depressive disorders, longitudinal study

## Abstract

**Background:**

Depression is a leading cause of healthcare use and risk for suicide among US military personnel. Depression is not well characterised over the shipboard deployment cycle, and personnel undergo less screening than with land-based deployments, making early identification less likely.

**Aims:**

To determine the demographic and behavioural risk factors associated with screening positive for risk of depression (ROD) across the shipboard deployment cycle.

**Method:**

Active-duty ship assigned personnel completed an anonymous assessment using the Center for Epidemiologic Studies Depression Scale (CES-D) in the year prior to deployment, during deployment and in the months following deployment. Longitudinal models were used to determine risk factors.

**Results:**

In total, 598 people were included in the analysis. Over 50% of the study population screened positive for ROD (CES-D score ≥16) and over 25% screened positive for risk of major depressive disorder (CES-D score ≥22) at all time points. Lower age, female gender, alcohol use, stress and prior mental health diagnoses were all associated with greater odds of screening positive for ROD in multivariable models.

**Conclusions:**

Although the risk factors associated with screening positive for ROD are similar to those in other military and civilian populations, the proportion screening positive exceeds previously reported prevalence. This suggests that shipboard deployment or factors associated with shipboard deployment may present particular stressors or increase the likelihood of depressive symptoms.

**Declaration of interest:**

The authors are military service members (or employees of the US Government). This work was prepared as part of the authors' official duties. Title 17, U.S.C. §105 provides the ‘Copyright protection under this title is not available for any work of the United States Government.’ Title 17, USC, §101 defines a US Government work as work prepared by a military service member or employee of the US Government as part of that person's official duties. The views expressed in this research are those of the authors and do not necessarily reflect the official policy or position of the Department of the Navy, Department of the Army, Department of the Air Force, Department of Veterans Affairs, Department of Defense, or the US Government. Approved for public release; distribution unlimited. Material has been reviewed by the Walter Reed Army Institute of Research. There is no objection to its presentation and/or publication. The opinions or assertions contained herein are the private views of the authors, and are not to be construed as official, or as reflecting true views of the Department of the Army or the Department of Defense.

Mental health disorders are a leading cause of healthcare use^1^ and medical evacuation^2^ (removal of an individual from a field or deployment scenario for the purpose of obtaining medical treatment) within the US military. Between 2007 and 2016, depressive disorders were the second most commonly diagnosed mental health disorder^3^ with 281 829 individuals diagnosed, comprising 16.8% of all mental health diagnoses in the US military. Without diagnosis and treatment, depression is associated with reduced productivity, lower likelihood of promotions, attrition and suicide.^4,5^ Suicide rates in the US military began rising in the mid-2000s and have exceeded civilian suicide rates since 2008.^6–9^ In both the US civilian and military populations, depression is associated with younger age, female gender, low educational attainment and income, and living alone; younger age and female gender being prominent risk factors.^3,10^ Following military service, 70% of military veterans will exclusively use civilian medical facilities,^11^ so civilian providers should be aware of these risk factors among this population.

Few data are available regarding depression across the shipboard deployment cycle. Recent data show that over 20% of personnel may be at risk for moderate-to-severe depression at the time of deployment.^12^ Shipboard personnel do not undergo a Pre-Deployment Health Assessment like their shore-based counterparts, so early identification may be reduced in this population. This study examines risk factors associated with screening positive for depression among US Navy and Marine Corps personnel in the 12 months before deployment, during deployment and 3 months after returning from a shipboard deployment.

## Method

Sampling, recruitment and data-collection methods were previously described in Harbertson *et al.*^13^ Briefly, longitudinal data were collected from US Navy and Marine Corps personnel via an anonymous, self-administered paper questionnaire between February 2012 and August 2014. Pre-deployment data were collected within 2 weeks of deployment; deployment data within the last 4 weeks of deployment; and post-deployment data 3 months after return from deployment (plus or minus 2 weeks). The pre-deployment questionnaire assessed behaviour in the previous 3 months, year, and ever; the deployment questionnaire assessed behaviour during the entire deployment; and the post-deployment questionnaire assessed behaviour since returning from deployment. The questionnaire included questions on demographics, sexual behaviours, alcohol use and mental health. For analysis, individuals were excluded if age or gender data were missing and were included if they completed the Center for Epidemiologic Studies Depression Scale (CES-D) at two or more time points.^14^

All questionnaires were completed anonymously. Questionnaires were linked using a series of eight linking questions,^15–17^ birth year, gender and ship number. Questionnaires with the same answer to 9 of the 11 questions we considered to be the same individual and were linked. The authors assert that all procedures contributing to this work comply with the ethical standards of the relevant national and institutional committees on human experimentation and with the Helsinki Declaration of 1975, as revised in 2008. All procedures involving human participants were approved by Naval Health Research Center Institutional Review Board (NHRC.2010.0033) and Walter Reed Army Institute of Research Human Subjects Protection Branch (WRAIR #1766). Written informed consent was obtained from all participants.

### Measures

The primary outcome was screening positive for depression. Scoring the CES-D included in the survey, individuals were classified as at risk for depression (ROD) with a score of 16 or higher and at risk of major depressive disorder (ROMDD) with a score of 22 or higher.^14,18^ Alcohol use was assessed using the Cut down, Annoyed, Guilty, Eye-opener (CAGE)^19^ questionnaire, the abbreviated Alcohol Use Disorders Identification Test (AUDIT-C),^20^ drinks per week/day/occasion and binge drinking. Individuals screened positive on the CAGE questionnaire if they answered yes to two or more questions, and they screened positive for dependent alcohol use on the AUDIT-C with a score of 8 or more. Drinks per week/day/occasion were used to classify individuals as heavy, moderate-heavy, moderate, light or non-drinkers using criteria from the Department of Defense Health Related Behaviors Survey (HRBS).^21^

If an individual reported being single in a committed relationship, single living with a partner, or married, they were categorised as being in a relationship. If an individual said they had previously been diagnosed with a depressive disorder, an anxiety disorder, post-traumatic stress disorder, combat stress reaction or a traumatic brain injury, they were categorised as having a previous diagnosis with a mental health condition of interest. A life stress inventory based on a modified Holmes-Rahe Stress Scale^22^ and HRBS was included. Because of the changes to the original scale, the inventory was not scored, but variables that indicated the presence of one or more stressor or the total number of stressors were included in the analysis.

### Data analysis

Descriptive statistics, including percentages, means and s.d. were calculated to describe the entire study population. All bivariate and multivariable analyses were conducted for the full population and for the population stratified by age or gender. Generalised estimating equations were used to calculate odds ratios (OR), 95% CI and *P*-values for these analyses, with *P*<0.05 considered statistically significant. A sensitivity analysis was conducted to compare the study population with the longitudinal cohort, all participants who completed more than one questionnaire, regardless of whether or not they completed the CES-D.

Demographics,^10,23^ mental health,^24^ alcohol use^25^ and substance misuse^26^ were considered risk factors analysed for their association with screening positive for depression. Variables were selected for inclusion in the final model based on their significance in the bivariate analysis. Variables with the highest *P*-values were eliminated manually using backwards elimination or replaced with another variable describing the same risk factor. All analyses were conducted using SAS software version 9.3 (SAS Institute Inc, Cary, NC, USA).

## Results

In total, 773 participants completed the survey at two or more time points. Two people were excluded because their age or gender data were missing, and 173 were excluded because they did not complete the CES-D portion of the questionnaire at two or more time points, leaving a final sample size of 598 available for analysis (Table 1). Sensitivity analysis found that the study population was not significantly different from the longitudinal cohort (data not shown). The majority were men (71.2%), White (59.3%) and had completed less than an undergraduate degree (84.4%). The average age was 26.3 years (s.d. = 6.5, median 24). Most were in one of two categories: ‘single, not in a committed relationship’ or ‘married’ (31.7 and 37.7%, respectively). Average length of service was 5.4 years (s.d. = 5.6, median  3) and 77.7% had completed at least one official deployment.

**Table 1:** Demographic characteristics of personnel responding to at least two questionnaires and completing the Center for Epidemiologic Studies Depression Scale

Characteristic	*n*	%	Mean	s.d.
Gender (*n* = 598)				
Men	426	71.2		
Women	172	28.8		
Age, years (*n* = 598)			26.3	6.5
17–20	83	13.9		
21–22	127	21.2		
23–24	101	16.9		
25–30	162	27.1		
31 and over	125	20.9		
Race and ethnicity (*n* = 594)				
White	352	59.3		
Black or African American	64	10.8		
Spanish/Hispanic/Latino	66	11.1		
Two or more races or ethnicities	45	7.6		
Other	67	11.3		
Marital status (*n* = 597)				
Single, uncommitted	189	31.7		
Single, committed relationship	110	18.4		
Single, living with partner	34	5.7		
Married	225	37.7		
Divorced, separated, or widowed	39	6.5		
Education level completed (*n* = 596)				
≤ High school, GED	237	39.8		
Some college, vocational	266	44.6		
≥ Undergraduate degree	93	15.6		
Service branch (*n* = 454)^a^				
Navy	425	93.6		
Marine Corps	29	6.4		
Military rank (*n* = 455)^a^				
E1–E3	164	36.0		
E4–E6	228	50.1		
E7–E9	23	5.1		
W1–W5, O1–O9	40	8.8		
Years in the military (*n* = 450)^a^			5.4	5.6
Number of official deployments (*n* = 580)^b^			1.8	2.1
0	129	22.2		
1	234	40.3		
≥2	217	37.4		

GED, General Education Development test

a. Data only collected pre-deployment.

b. Data collected pre- and post-deployment; post-deployment information used for those without pre-deployment information.

**Fig. 1:**
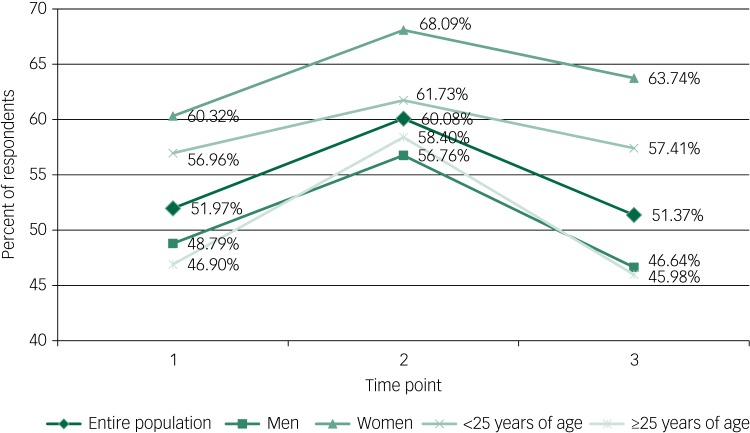
Percent of respondents at risk for depression by time point.

During deployment, a greater proportion of respondents screened positive for ROD (60.1 *v.* 52.0% at *T*_1_ and 51.4% at *T*_3_; *P* = 0.01, *P* = 0.01) and ROMDD (32.4 *v.* 27.2% at *T*_1_ and 26.4% at *T*_3_; *P* = 0.06, *P* = 0.07) (Fig. 1 and supplementary Fig. 1 available at https://doi.org/10.1192/bjo.2019.70). A significantly greater proportion of women and respondents under 25 years old screened positive for ROMDD and ROD than those over 25 and men.

### Bivariate analysis

In unadjusted analysis, younger age and female gender were associated with screening positive for ROD (supplementary Table 1). Other factors associated with increased odds of screening positive for ROD included Hispanic/Latino ethnicity, screening positive for alcohol misuse, prior mental health conditions or having a recent stressful life experience in both the combined and stratified study populations. Lower odds of screening positive for ROD were observed among those reporting an undergraduate degree or higher, or officer or warrant officer rank in the combined study population as well as in the male-only and 25 year and over stratified analyses. Reporting being in a relationship or deploying one or more times were also associated with significantly lower odds of screening positive for ROD in the combined study population.

### Multivariable analysis

Many factors associated with screening positive for ROD remained significant in multivariable regressions (Table 2, supplementary Tables 2–5). For the entire study population, older age was associated with lower odds of screening positive for ROD (OR = 0.95, 95% CI 0.91–0.98). Female gender (OR = 1.61 95% CI 1.05–2.46), prior diagnosis with a mental health condition of interest (OR = 2.58, 95% CI 1.42–4.65) and experiencing at least one stressful event (OR = 1.82, 95% CI 1.21–2.74) were significantly associated with screening positive for ROD (Table 2). Screening positive on the CAGE (OR = 1.80, 95% CI 1.05–3.08) and moderate-heavy to heavy drinking (OR = 1.47, 95% CI 1.03–2.11) had a slightly weaker association but were still significantly associated with ROD.

**Table 2:** Longitudinal model describing risk factors for screening positive for depression among all respondents

	OR (95% CI)	*P*
Age	**0.95 (0.91–0.98)**	**0.006**
Gender, women	**1.61 (1.05–2.46)**	**0.027**
Relationship status		
In a relationship	0.85 (0.56–1.29)	0.444
Longest amount of time away from partner		
1 month or less (referent)	N/A	N/A
Greater than 1 month	1.15 (0.73–1.81)	0.546
Race or ethnicity		
White (referent)	N/A	N/A
Black	1.17 (0.64–2.02)	0.652
Hispanic	**1.98 (1.12–3.50)**	**0.019**
Other	1.54 (0.93–2.54)	0.095
Rank		
Enlisted (referent)	N/A	N/A
W1–W5, O1–O9	0.91 (0.48–1.73)	0.783
Military experience		
No deployments (referent)	N/A	N/A
1 deployment	1.07 (0.62–1.84)	0.819
2 or more deployments	0.92 (0.49–1.73)	0.793
Alcohol		
Positive CAGE screening	**1.80 (1.05–3.08)**	**0.032**
Lighter than moderate-heavy drinker (referent)	N/A	N/A
Moderate-heavy to heavy drinker	**1.47 (1.03–2.11)**	**0.034**
Have ever passed out/blacked out from drinking	1.03 (0.70–1.52)	0.881
Mental health		
Any mental health condition of interest	**2.58 (1.42–4.65)**	**0.002**
Stress		
At least one stressful event	**1.82 (1.21–2.74)**	**0.004**
Drug use		
Have ever used any drugs	0.54 (0.28–1.04)	0.067

Results in bold are significant.

N/A, not applicable; CAGE, Cut down, Annoyed, Guilty, Eye-opener.

Among female participants (supplementary Table 2), being in the 21–22 and 23–24 year old age groups (OR = 11.98 (95% CI 2.91–49.36) and 8.18 (95% CI 2.13–31.35), respectively), experiencing at least one stressful event (OR = 3.59, 95% CI 1.54–8.34), and reporting Black race (OR = 2.78, 95% CI 1.22–6.30), one (OR = 3.24, 95% CI 1.33–7.88) or two or more (OR = 4.56, 95% CI 1.77–11.77) previous deployments, and prior diagnosis with an anxiety disorder (OR = 2.72, 95% CI 1.09–6.80) were significantly associated with screening positive for ROD.

Among male respondents (supplementary Table 3), consuming alcohol in the previous 12 months (OR = 3.11, 95% CI 1.43–6.80), screening positive on the CAGE (OR = 2.79, 95% CI 1.49–5.24) and prior diagnosis with a mental health condition of interest (OR = 3.11, 95% CI 1.43–6.80) were associated with screening positive for ROD. Although there was no association with having a recent stressful experience, those with more stressful experiences were somewhat more likely to screen positive for ROD (OR = 1.18, 95% CI 1.08–1.29). Those who were single but living with a partner (OR = 0.32, 95% CI 0.13–0.76) or married (OR = 0.46, 95% CI 0.24–0.89) had lower odds of screening positive for ROD.

Among those aged 24 years and younger (supplementary Table 4), being female (OR = 3.17, 95% CI 1.65–6.09) and having a recent stressful experience (OR = 2.36, 95% CI 1.28–4.38) were more likely to screen positive for ROD. The association between moderate-heavy to heavy drinking (OR = 1.71, 95% CI 1.00–2.90) and likelihood to screen positive for ROD was significant but weaker than the other associations.

Among participants ≥25 years of age (supplementary Table 5), participants who were Hispanic/Latino (OR = 2.52, 95% CI 1.02–6.24) or had a prior diagnosis with a mental health condition of interest (OR = 3.86, 95% CI 1.67–8.91) had higher odds of screening positive for ROD. Those who had experienced more stressful events also had somewhat higher odds of screening positive for ROD (OR = 1.20, 95% CI 1.08–1.33). Those who were single but living with a partner (OR = 0.29, 95% CI 0.09–0.97), married (OR = 0.35, 95% CI 0.14–0.86), or divorced, separated, or widowed (OR = 0.22, 95% CI 0.06–0.83) were less likely to screen positive for ROD than those who were single.

## Discussion

Younger personnel, women, people who screened positive for alcohol misuse, people previously diagnosed with a mental health condition of interest and those who had experienced one or more stressful events had higher odds of screening positive for ROD. Black race or Hispanic ethnicity and being single was associated with screening positive for ROD in women and those 25 years or older. Among women only, more deployments were associated with screening positive for ROD.

A substantial proportion of participants screened positive for ROD or ROMDD, with the highest prevalence reported during deployment. The prevalence of screening positive for ROD or ROMDD is higher than observed in previous studies among US military personnel.^4,5,27,28^ These previous studies were generally conducted immediately post-deployment and used different criteria for determining ROD. Between May 2003 and April 2004, 4.5% of US Army and Marines personnel returning from Operation Iraqi Freedom (OIF) gave one positive response on the Patient Health Questionnaire-2, and 19.1% had any mental health concern.^4^ A longitudinal study of enlisted personnel returning from OIF beginning in 2003 found that 36.5% of respondents had a score between 9 and 26 on the CES-D 12 months post-deployment.^28^ From October 2009 to March 2013, 7% of all respondents completing their first Pre-Deployment Health Assessment screened positive for ROD.^27^ Among non-deployed Army personnel in 2011, 4.8% were found to have ROMDD in the previous 30 days.^5^ The highest proportion among these studies, 36.5%, was obtained using less stringent scoring requirements than our study and remains substantially lower than the 50.1% screening positive for ROD post-deployment, the most comparable time point. The characteristics associated with screening positive for ROD within this population, such as younger age and female gender, mirror those in the broader US military population and the US civilian population,^3,29^ but the proportion of this study's population screening positive for depression is much higher. The lowest prevalence for the entire study population was 51.4% at *T*_3_ whereas only 8.1% of the entire US population over 20 screened positive for a moderate ROD between 2013 and 2016.^30^

This discrepancy could be caused by a number of factors. The study population is younger and has a higher proportion of women than the general US military and US Navy populations.^31^ However, the proportion of men and individuals aged 25 years and older screening positive for ROD were still higher when compared with studies among the US military or civilian populations.^4,5,27–29^ Screening studies among US college students have found the proportion of students screening positive for ROD to be between 23.5 and 38.5%.^32–34^ Among study participants under 25 years of age, 57% screened positive for ROD at *T*_1_. The difference may be because of unmeasured factors or quite possibly the deployment itself, particularly given the stress associated with a sudden change of status. That the proportion screening positive for ROD increased dramatically during deployment and returned to pre-deployment levels after returning supports the idea that deployment may contribute to ROD. Although similar stresses to deployment may be experienced by first-year college students, few studies stratified their results by student year. In the one that did, a higher proportion of first years screened positive for ROD than did other years, although this proportion (34.9%) was still lower than that of this study's population.^32^ A study of shipboard military personnel in the UK found that they also reported mental health issues more frequently than personnel on land-based deployments,^35^ which further suggests that shipboard deployments themselves may contribute to screening positive for ROD.

In stratified analysis, women with one or more deployments, prior anxiety disorder or identification as Black race were associated with higher prevalence of screening positive for ROD. In addition, among those 25 years of age or older, identification of Hispanic/Latino status was also associated with ROD. In military populations, socioeconomic status is largely determined by rank, although there are complex relationships between rank and other variables, including race and ethnicity.^31^ However, non-socioeconomic factors, including differential attitudes towards mental health and care seeking or social stresses, may also contribute to the association of race and ethnicity and depression in this population. Prior deployment history or prior anxiety disorder history could serve as screening questions to identify higher risk women for appropriate treatment.

Screening positive on the CAGE questionnaire (men) or moderate/heavy drinking (under 25) was associated with screening positive for ROD. The CAGE questionnaire is intended to detect alcoholism, which is strongly associated with ROD as both a potential cause and symptom.^25^ Alcohol misuse is associated with a variety of issues of concern to the military and civilian community (motor vehicle accidents, productivity loss, dependency and many others)^36^ so targeting these groups for alcohol use interventions could result in health improvements beyond depression risk. Similarly, because the association between depression and alcohol is bidirectional, reducing ROD could result in less alcohol use with similar benefits. Also, the association between prior depression diagnosis and current screen positive for ROD could be used to screen for high-risk men.

### Strengths and limitations

Although there was a sizeable proportion of individuals who did not complete their CES-D screen, the anonymous voluntary survey likely encouraged truthful reporting. However, the absence of identifiers may have led to missed or incorrect linkage, reducing or biasing the longitudinal study population and limiting our ability to detect correct associations. We used convenience sampling techniques to capture the greatest number of participants, and logistic constraints did not allow universal enrolment on each ship. However, we approached and recruited participants from every department, with intent to generate a diverse sample. Approximately one-quarter of the individuals in the linked data-set did not complete the CES-D, which could generate bias. However, the group who completed the CES-D is demographically very similar to the entire study population.

The CES-D has comparable diagnostic validity to other depression screening tools.^36^ The cut-off score of 16 may identify false positives,^37^ but inclusion of the major depressive disorder screening likely improves specificity. The entire study population has a greater proportion of personnel under 25 and women than the US Navy or US military at large, which are high-risk groups for depression.^31^ Although this affects generalizability to other populations, it is still likely generalisable to other shipboard populations and may apply to other services across any deployment or similar young civilian populations undergoing prolonged sequestration, such as dormitory college students.

### Implications

The large proportion of people screening positive for ROD merits further attention. The numbers and associations found regarding depression over the shipboard deployment cycle highlight a need for mental health services. A return to a pre-deployment proportion screening positive for ROD following deployment suggests that deployment may not have a permanent effect on mental health. However, the pre- and post-deployment proportions screening positive for ROD are still higher than seen in comparable military population in similar circumstances.^2,27,28^ Even if the mental health effects from deployment are temporary, addressing mental health concerns during deployment are still necessary for this population's overall health and well-being. More intensive mental health resources could be targeted to high-risk groups throughout the deployment cycle. Interventions shown to be effective among land-based deploying military personnel, such as secondary screening to ensure that individuals on a psychotherapeutic medication are on a stable dose and have enough doses for the duration of the deployment, could be added to pre-deployment screenings for high-risk groups before shipboard deployment.^38^ One-on-one interventions, such as cognitive–behavioural therapy, are logistically unrealistic during a shipboard deployment; however, telemedicine sessions could be intermittently available to continue or initiate care with land-based providers.^39^ In the military, leadership behaviour to encourage social engagement and target faulty perceptions of isolation have been shown to improve unit cohesion.^28^ Other programmes such as group cognitive–behavioural therapy, exercise and mindfulness have demonstrated some effectiveness at treating or preventing the onset of depressive symptoms.^40–42^ Further research is needed to assess the effectiveness and feasibility of such interventions in a shipboard deployment cycle.

In conclusion, this study found that, although the risk factors associated with screening positive for ROD were similar to civilian and other military populations, the proportion of military personnel that screened positive for ROD throughout the shipboard deployment cycle exceeded prevalence reported among other military populations and civilians. This suggests that shipboard deployment presents particular stressors to mental health or that unknown factors associated with shipboard deployment may increase likelihood of depressive symptoms. Future studies should explore the benefit of adding a brief mental health screening^37^ or including the pre-deployment health assessment survey for shipboard populations to identify those most at risk for depression and ensure they have adequate medication plans and counselling support during deployment. Implementation of evidence-based interventions feasible in such isolated settings as described previously could be explored in follow-on studies.

## Supplementary material

For supplementary material accompanying this paper visit http://dx.doi.org/10.1192/bjo.2019.70. click here to view supplementary material

## References

[1] Armed Forces Health Surveillance Branch. Absolute and relative morbidity burdens attributable to various illnesses and injuries, active component, U.S. Armed Forces, 2016. MSMR 2017; 24: 2–8.

[2] Cohen SP, Brown C, Kurihara C, Plunkett A, Nguyen C, Strassels SA. Diagnoses and factors associated with medical evacuation and return to duty for service members participating in Operation Iraqi Freedom or Operation Enduring Freedom: a prospective cohort study. Lancet 2010; 375: 301–9. 20109957 10.1016/S0140-6736(09)61797-9

[3] Stahlman S, Oetting AA. Mental health disorders and mental health problems, active component, U.S. Armed Forces, 2007-2016. MSMR 2018; 25: 2–11. 29578729

[4] Hoge CW, Auchterlonie JL, Milliken CS. Mental health problems, use of mental health services, and attrition from military service after returning from deployment to Iraq or Afghanistan. JAMA 2006; 295: 1023–32. 16507803 10.1001/jama.295.9.1023

[5] Kessler RC, Heeringa SG, Stein MB, Colpe LJ, Fullerton CS, Hwang I, Thirty-day prevalence of DSM-IV mental disorders among nondeployed soldiers in the US Army: results from the Army Study to Assess Risk and Resilience in Servicemembers (Army STARRS). JAMA Psychiatry 2014; 71: 504–13. 24590120 10.1001/jamapsychiatry.2014.28 PMC4057988

[6] Kuehn BM. Soldier suicide rates continue to rise: military, scientists work to stem the tide. JAMA 2009; 301: 1112–13. 10.1001/jama.2009.342 19293405

[7] Nock MK, Deming CA, Fullerton CS, Gilman SE, Goldenberg M, Kessler RC, Suicide among soldiers: a review of psychosocial risk and protective factors. Psychiatry 2013; 76: 97–125. 23631542 10.1521/psyc.2013.76.2.97 PMC4060831

[8] Pruitt LD, Smolenski DJ, Bush NE, Skopp NA, Hoyt TV, Grady BJ. Department of Defense Suicide Event Report: Calendar Year 2015 Annual Report: 135 Publication No. E-6A4ED71 National Center for Telehealth & Technology, Defense Centers of Excellence for Psychological Health and Traumatic Brain Injury, 2017.

[9] Reger MA, Pruitt LD, Smolenski DJ. Lessons from the latest US military suicide surveillance data. J Clin Psychiatry 2018; 79: 17l11790. 10.4088/JCP.17l11790 29505185

[10] Bromet E, Andrade LH, Hwang I, Sampson NA, Alonso J, de Girolamo G, Cross-national epidemiology of DSM-IV major depressive episode. BMC Med 2011; 9: 90. 21791035 10.1186/1741-7015-9-90 PMC3163615

[11] US Department of Veterans Affairs. Profile of Veterans: 2013. US Department of Veterans Affairs, 2015 (https://www.va.gov/vetdata/docs/SpecialReports/Profile_of_Veterans_2013.pdf ).

[12] Harbertson J, Hale BR, Michael NL, Scott PT. Missed opportunity to screen and diagnose PTSD and depression among deploying shipboard US military personnel. BJPsych Open 2016; 2: 314–17. 27713833 10.1192/bjpo.bp.116.003038 PMC5051555

[13] Harbertson J, Scott PT, Moore J, Wolf M, Morris J, Thrasher S, Sexually transmitted infections and sexual behaviour of deploying shipboard US military personnel: a cross-sectional analysis. Sex Transm Infect 2015; 91: 581–8. 26586849 10.1136/sextrans-2015-052163

[14] Radloff LS. The CES-D Scale: a self-report depression scale for research in the general population. Appl Psychol Meas 1977; 1: 385–401.

[15] Grube JW, Morgan M, Kearney KA. Using self-generated identification codes to match questionnaires in panel studies of adolescent substance use. Addict Behav 1989; 14: 159–71. 2786325 10.1016/0306-4603(89)90044-0

[16] Honig F. When you can't ask their names: linking anonymous respondents with the Hogben number. Aust J Public Health 1995; 19: 94–6. 7734604 10.1111/j.1753-6405.1995.tb00305.x

[17] DiIorio C, Soet JE, Van Marter D, Woodring TM, Dudley WN. An evaluation of a self-generated identification code. Res Nurs Health 2000; 23: 167–74. 10782875 10.1002/(sici)1098-240x(200004)23:2<167::aid-nur9>3.0.co;2-k

[18] Klinkman MS, Coyne JC, Gallo S, Schwenk TL. Can case-finding instruments be used to improve physician detection of depression in primary care? Arch Fam Med 1997; 6: 567–73. 9371051 10.1001/archfami.6.6.567

[19] O'Brien CP. The CAGE questionnaire for detection of alcoholism. JAMA 2008; 300: 2054–6. 18984895 10.1001/jama.2008.570

[20] Bush K, Kivlahan DR, McDonell MB, Fihn SD, Bradley KA. The AUDIT alcohol consumption questions (AUDIT-C): an effective brief screening test for problem drinking. Ambulatory Care Quality Improvement Project (ACQUIP). Alcohol Use Disorders Identification Test. Arch Intern Med 1998; 158: 1789–95. 9738608 10.1001/archinte.158.16.1789

[21] RTI International. Department of Defense Survey of Health Related Behaviors Among Active Duty Military Personnel: A Component of the Defense Lifestyle Assessment Program (DLAP): 678 RTI International, 2009.

[22] Holmes TH, Rahe RH. The social readjustment rating scale. J Psychosom Res 1967; 11: 213–8. 6059863 10.1016/0022-3999(67)90010-4

[23] Rao D, Feinglass J, Corrigan P. Racial and ethnic disparities in mental illness stigma. J Nerv Ment Dis 2007; 195: 1020–3. 18091196 10.1097/NMD.0b013e31815c046e

[24] Kessler RC, Berglund P, Demler O, Jin R, Koretz D, Merikangas KR, The epidemiology of major depressive disorder: results from the National Comorbidity Survey Replication (NCS-R). JAMA 2003; 289: 3095–105. 12813115 10.1001/jama.289.23.3095

[25] Grant BF, Harford TC. Comorbidity between DSM-IV alcohol use disorders and major depression: results of a national survey. Drug Alcohol Depend 1995; 39: 197–206. 8556968 10.1016/0376-8716(95)01160-4

[26] Grant BF. Comorbidity between DSM-IV drug use disorders and major depression: results of a national survey of adults. J Subst Abuse 1995; 7: 481–97. 8838629 10.1016/0899-3289(95)90017-9

[27] Shen YC, Arkes J, Lester PB. Association between baseline psychological attributes and mental health outcomes after soldiers returned from deployment. BMC Psychol 2017; 5: 32. 28978357 10.1186/s40359-017-0201-4 PMC5628451

[28] Vasterling JJ, Proctor SP, Aslan M, Ko J, Jakupcak M, Harte CB, Military, demographic, and psychosocial predictors of military retention in enlisted army soldiers 12 months after deployment to Iraq. Mil Med 2015; 180: 524–32. 25939106 10.7205/MILMED-D-14-00468

[29] Hasin DS, Sarvet AL, Meyers JL, Saha TD, Ruan WJ, Stohl M, Epidemiology of adult DSM-5 major depressive disorder and its specifiers in the United States. JAMA Psychiatry 2018; 75: 336–46. 29450462 10.1001/jamapsychiatry.2017.4602 PMC5875313

[30] Brody DJ, Pratt LA, Hughes J. Prevalence of Depression Among Adults Aged 20 and Over: United States, 2013–2016: 8 NCHS Data Brief, no 303 National Center for Health Statistics, 2018 (https://www.cdc.gov/nchs/products/databriefs/db303.htm). 29638213

[31] US Department of Defense. 2016 Demographics: Profile of the Military Community. US Department of Defense, no date (http://download.militaryonesource.mil/12038/MOS/Reports/2016-Demographics-Report.pdf).

[32] Wilson KT, Bohnert AE, Ambrose A, Davis DY, Jones DM, Magee MJ. Social, behavioral, and sleep characteristics associated with depression symptoms among undergraduate students at a women's college: a cross-sectional depression survey, 2012. BMC Womens Health 2014; 14: 8. 24410897 10.1186/1472-6874-14-8 PMC3893578

[33] Herman S, Archambeau OG, Deliramich AN, Kim BS, Chiu PH, Frueh BC. Depressive symptoms and mental health treatment in an ethnoracially diverse college student sample. J Am Coll Health 2011; 59: 715–20. 21950252 10.1080/07448481.2010.529625 PMC3210726

[34] Wolanin A, Hong E, Marks D, Panchoo K, Gross M. Prevalence of clinically elevated depressive symptoms in college athletes and differences by gender and sport. Br J Sports Med 2016; 50: 167–71. 26782764 10.1136/bjsports-2015-095756

[35] Whybrow D, Jones N, Evans C, Minshall D, Smith D, Greenberg N. The mental health of deployed UK maritime forces. Occup Environ Med 2016; 73: 75–82. 26265671 10.1136/oemed-2015-102961 PMC4752642

[36] Brown JM, Bray RM, Hartzell MC. A comparison of alcohol use and related problems among women and men in the military. Mil Med 2010; 175: 101–7. 20180479 10.7205/milmed-d-09-00080

[37] Shean G, Baldwin G. Sensitivity and specificity of depression questionnaires in a college-age sample. J Genet Psychol 2008; 169: 281–8. 18788328 10.3200/GNTP.169.3.281-292

[38] Warner CH, Appenzeller GN, Parker JR, Warner CM, Hoge CW. Effectiveness of mental health screening and coordination of in-theater care prior to deployment to Iraq: a cohort study. Am J Psychiatry 2011; 168: 378–85. 21245086 10.1176/appi.ajp.2010.10091303

[39] Hilty DM, Ferrer DC, Parish MB, Johnston B, Callahan EJ, Yellowlees PM. The effectiveness of telemental health: a 2013 review. Telemed J E Health 2013; 19: 444–54. 23697504 10.1089/tmj.2013.0075 PMC3662387

[40] Resick PA, Wachen JS, Dondanville KA, Pruiksma KE, Yarvis JS, Peterson AL, Effect of group vs individual cognitive processing therapy in active-duty military seeking treatment for posttraumatic stress disorder: a randomized clinical trial. JAMA Psychiatry 2017; 74: 28–36. 27893032 10.1001/jamapsychiatry.2016.2729

[41] Ströhle A. Physical activity, exercise, depression and anxiety disorders. J Neural Transm (Vienna) 2009; 116: 777–84. 18726137 10.1007/s00702-008-0092-x

[42] Farb N, Anderson A, Ravindran A, Hawley L, Irving J, Mancuso E, Prevention of relapse/recurrence in major depressive disorder with either mindfulness-based cognitive therapy or cognitive therapy. J Consult Clin Psychol 2018; 86: 200–4. 29265831 10.1037/ccp0000266

