# Supplementary material for: Risk factors for positive depression screening across a shipboard deployment cycle
Source: BJPsych Open. 2019 Sep 20;5(5):e84. doi: 10.1192/bjo.2019.70 (PMC6788222; doi:10.1192/bjo.2019.70)
Supplement: Supplementary file 1 [file S205647241900070Xsup001.zip › S205647241900070Xsup001/Supp Table 1.docx]

| Supplementary Table 1. Demographic and behavioral characteristics associated with being at risk for depression | | | | | | | | | | | | | | | | | | | |  |
| --- | --- | --- | --- | --- | --- | --- | --- | --- | --- | --- | --- | --- | --- | --- | --- | --- | --- | --- | --- | --- |
|  | Full | | | | Men | | | | Women | | | | 24 and under | | | 25 and over | | | |  |
| Characteristic | OR  (95% CI) | | p-value* | | OR  (95% CI) | | p-value* | | OR  (95% CI) | | p-value* | | OR  (95% CI) | | p-value* | OR  (95% CI) | | p-value* | |  |
| Gender |  | |  | |  | |  | |  | |  | |  | |  |  | |  | |  |
| Male (referent) |  | |  | |  | |  | |  | |  | |  | |  |  | |  | |  |
| Female | **1.70 (1.27, 2.27)** | | **<0.001** | | N/A | | N/A | | N/A | | N/A | | **1.89 (1.28, 2.79)** | | **0.001** | 1.37 (0.89, 2.13) | | 0.153 | |  |
| Age | **0.97 (0.95, 0.99)** | | **<0.001** | | **0.98 (0.95, 1.00)** | | **0.044** | | **0.95 (0.92, 0.99)** | | **0.015** | | 1.06 (0.94, 1.19) | | 0.317 | **0.96 (0.93, 0.98)** | | **0.003** | |  |
| 17-20 | 1.07 (0.72, 1.59) | | 0.729 | | 1.36 (0.83, 2.23) | | 0.220 | | 0.60 (0.31, 1.16) | | 0.129 | | 0.89 (0.58, 1.35) | | 0.572 | N/A | | N/A | |  |
| 21-22 | 1.18 (0.88, 1.59) | | 0.268 | | 1.01 (0.71, 1.45) | | 0.941 | | 1.43 (0.83, 2.48) | | 0.202 | | 0.97 (0.69, 1.38) | | 0.882 | N/A | | N/A | |  |
| 23-24 | 1.27 (0.91,1.77) | | 0.155 | | 1.05 (0.71, 1.56) | | 0.804 | | **2.02 (1.05, 3.91)** | | **0.037** | | 1.13 (0.78, 1.64) | | 0.516 | N/A | | N/A | |  |
| 25-30 | 1.01 (0.77, 1.34) | | 0.928 | | 1.06 (0.77, 1.48) | | 0.712 | | 0.88 (0.52, 1.48) | | 0.624 | | N/A | | N/A | **1.49 (1.03, 2.14)** | | **0.034** | |  |
| 31 and over | **0.63 (0.46, 0.86)** | | **0.004** | | 0.75 (0.53, 1.07) | | 0.113 | | **0.41 (0.18, 0.93)** | | **0.03** | | N/A | | N/A | **0.67 (0.47, 0.97)** | | **0.034** | |  |
| Race/ethnicity |  | |  | |  | |  | |  | |  | |  | |  |  | |  | |  |
| White | 0.95 (0.73, 1.24) | | 0.704 | | 0.92 (0.67, 1.26) | | 0.585 | | 1.15 (0.69, 1.91) | | 0.597 | | 1.03 (0.69, 1.53) | | 0.881 | 0.82 (0.57, 1.19) | | 0.297 | |  |
| Black or African American | 1.22 (0.84, 1.78) | | 0.305 | | 0.988 (0.61, 1.59) | | 0.961 | | 1.59 (0.84, 2.98) | | 0.149 | | 0.98 (0.58, 1.65) | | 0.932 | 1.53 (0.89, 2.64) | | 0.127 | |  |
| Spanish/Hispanic/ Latino | **1.52 (1.03, 2.24)** | | **0.035** | | 1.46 (0.90, 2.35) | | 0.124 | | 1.43 (0.73, 2.79) | | 0.298 | | 1.33 (0.78, 2.25) | | 0.296 | 1.71 (0.98, 3.02) | | 0.060 | |  |
| Other | 1.10 (0.77, 1.56) | | 0.605 | | 1.27 (0.84, 1.91) | | 0.265 | | 0.76 (0.39, 1.46) | | 0.410 | | **1.78 (1.01, 3.15)** | | **0.048** | 0.83 (0.53, 1.32) | | 0.431 | |  |
| Marital status |  | |  | |  | |  | |  | |  | |  | |  |  | |  | |  |
| Single, uncommitted | 1.27 (0.97, 1.66) | | 0.076 | | 1.26 (0.92, 1.73) | | 0.163 | | 1.27 (0.78, 2.05) | | 0.338 | | 1.03 (0.73, 1.46) | | 0.862 | 1.49 (0.96, 2.31) | | 0.0750 | |  |
| Single, committed relationship | 0.91 (0.67, 1.25) | | 0.567 | | 0.98 (0.66, 1.44) | | 0.900 | | 0.69 (0.40, 1.18) | | 0.179 | | 0.71 (0.48, 1.05) | | 0.089 | 1.21 (0.68, 2.13) | | 0.521 | |  |
| Single, living with partner | 1.10 (0.68, 1.78) | | 0.700 | | 0.83 (0.47, 1.44) | | 0.502 | | 1.26 (0.54, 2.93) | | 0.589 | | 1.44 (0.73, 2.83) | | 0.294 | 0.87 (0.42, 1.78) | | 0.700 | |  |
| Married | 0.77 (0.60, 1.01) | | 0.061 | | 0.84 (0.62, 1.14) | | 0.258 | | 0.93 (0.50, 1.73) | | 0.813 | | 1.05 (0.67, 1.63) | | 0.837 | 0.76 (0.53, 1.09) | | 0.132 | |  |
| Divorced, separated, or widowed | 1.17 (0.71, 1.92) | | 0.529 | | 1.07 (0.58, 2.00) | | 0.823 | | 1.09 (0.47, 2.53) | | 0.840 | | 2.88 (0.77, 10.75) | | 0.116 | 0.99 (0.56, 1.74) | | 0.973 | |  |
| In a relationship | **0.77 (0.60, 0.99)** | | **0.05** | | 0.80 (0.59, 1.09) | | 0.155 | | 0.79 (0.50, 1.25) | | 0.312 | | 0.86 (0.61, 1.21) | | 0.389 | 0.77 (0.53, 1.13) | | 0.182 | |  |
| Education level completed |  | |  | |  | |  | |  | |  | |  | |  |  | |  | |  |
| < High school, GED | 0.97 (0.75, 1.25) | | 0.811 | | 1.06 (0.78, 1.43) | | 0.710 | | 0.77 (0.48, 1.23) | | 0.271 | | 0.85 (0.59, 1.21) | | 0.368 | 0.77 (0.50, 1.19) | | 0.235 | |  |
| Some college, vocational | **1.30 (1.01, 1.66)** | | **0.042** | | 1.29 (0.97, 1.73) | | 0.100 | | 1.33 (0.83, 2.15) | | 0.233 | | 1.08 (0.75, 1.56) | | 0.661 | **1.76 (1.23, 2.51)** | | **0.002** | |  |
| > Undergraduate degree | **0.65 (0.45, 0.93)** | | **0.020** | | **0.57 (0.38, 0.85)** | | **0.006** | | 0.92 (0.43, 1.98) | | 0.841 | | 1.88 (0.55, 6.40) | | 0.310 | **0.60 (0.40, 0.91)** | | **0.016** | |  |
| Longest time away from partner |  | |  | |  | |  | |  | |  | |  | |  |  | |  | |  |
| 1 month or less | 0.83 (0.62, 1.12) | | 0.230 | | 0.80 (0.56, 1.13) | | 0.205 | | 0.97 (0.51, 1.83) | | 0.920 | | 1.23 (0.76, 2.00) | | 0.392 | 0.70 (0.46, 1.04) | | 0.080 | |  |
| Greater than 1 month | 1.07 (0.75, 1.53) | | 0.693 | | 1.19 (0.78, 1.81) | | 0.413 | | 0.87 (0.45, 1.67) | | 0.688 | | 1.07 (0.63, 1.84) | | 0.794 | 1.10 (0.68, 1.78) | | 0.698 | |  |
| Military rank |  | |  | |  | |  | |  | |  | |  | |  |  | |  | |  |
| Enlisted (referent) |  | |  | |  | |  | |  | |  | |  | |  |  | |  | |  |
| W1–W5, O1–O9 | **0.54 (0.35, 0.97)** | | **0.034** | | **0.37 (0.21, 0.66)** | | **<0.001** | | 1.00 (0.38, 2.64) | | 0.994 | | 1.84 (0.47, 7.29) | | 0.383 | **0.47 (0.26, 0.83)** | | **0.010** | |  |
| Number of official deployments | **0.93 (0.88, 0.98)** | | **0.008** | | 0.95 (0.89, 1.01) | | 0.116 | | 0.97 (0.83, 1.14) | | 0.736 | | 0.95 (0.80, 1.12) | | 0.524 | 0.96 (0.90, 1.03) | | 0.247 | |  |
| 0 | 1.07 (0.76, 1.52) | | 0.686 | | 1.37 (0.89, 2.09) | | 0.149 | | 0.63 (0.35, 1.13) | | 0.120 | | 1.09 (0.74, 1.60) | | 0.679 | 0.78 (0.37, 1.63) | | 0.501 | |  |
| 1 | 1.10 (0.88, 1.37) | | 0.391 | | 0.98 (0.75, 1.28) | | 0.875 | | 1.25 (0.82, 1.90) | | 0.292 | | 0.88 (0.66, 1.18) | | 0.393 | 1.25 (0.86, 1.82) | | 0.248 | |  |
| 2 or more | 0.87 (0.68, 1.10) | | 0.233 | | 0.89 (0.67, 1.19) | | 0.433 | | 1.02 (0.63, 1.64) | | 0.938 | | 1.12 (0.77, 1.63) | | 0.536 | 0.86 (0.61, 1.23) | | 0.412 | |  |
| Alcohol Use | |  | |  | |  | |  | |  | |  | |  |  | |  | |  | |
| Positive CAGE screening | | **2.18 (1.52, 3.12)** | | **<0.001** | | **2.76 (1.82, 4.18)** | | **<0.001** | | 1.14 (0.57, 2.27) | | 0.707 | | **2.62 (1.48, 4.64)** | **0.001** | | **1.95 (1.20, 3.19)** | | **0.007** | |
|  | |  | |  | |  | |  | |  | |  | |  |  | |  | |  | |
| Moderate-heavy to heavy drinker | | **1.37 (1.09, 1.70)** | | **0.006** | | **1.37 (1.05, 1.79)** | | **0.019** | | **1.54 (1.01, 2.34)** | | **0.046** | | **1.73 (1.25, 2.39)** | **<0.001** | | 1.11 (0.81, 1.51) | | 0.517 | |
|  | |  | |  | |  | |  | |  | |  | |  |  | |  | |  | |
| AUDIT-C score: ≥4 women, ≥5 men | | **1.34 (1.04, 1.71)** | | **0.02** | | **1.42 (1.06, 1.89)** | | **0.017** | | 1.21 (0.74, 1.96) | | 0.446 | | **1.43 (1.00, 2.03)** | **0.048** | | 1.25 (0.87, 1.78) | | 0.230 | |
|  | |  | |  | |  | |  | |  | |  | |  |  | |  | |  | |
| Have ever passed out/blacked out from drinking | | 1.14 (0.89, 1.46) | | 0.284 | | 1.11 (0.83, 1.47) | | 0.485 | | 1.34 (0.80, 2.25) | | 0.266 | | **1.54 (1.07, 2.23)** | **0.021** | | 0.87 (0.62, 1.22) | | 0.426 | |
|  | |  | |  | |  | |  | |  | |  | |  |  | |  | |  | |
| Have consumed alcohol in the past year | | **1.77 (1.26, 2.48)** | | **<0.001** | | **1.69 (1.12, 2.56)** | | **0.013** | | **1.96 (1.10, 3.48)** | | **0.022** | | **2.07 (1.35, 3.15)** | **<0.001** | | 1.50 (0.87, 2.61) | | 0.148 | |
|  | |  | |  | |  | |  | |  | |  | |  |  | |  | |  | |
| Drug use | |  | |  | |  | |  | |  | |  | |  |  | |  | |  | |
| Have ever used any drugs | | 0.73 (0.44, 1.21) | | 0.220 | | 0.94 (0.51, 1.72) | | 0.841 | | 0.40 (0.15, 1.02) | | 0.055 | | 0.69 (0.33, 1.44) | 0.321 | | 0.77 (0.37, 1.61) | | 0.493 | |
|  | |  | |  | |  | |  | |  | |  | |  |  | |  | |  | |
| Mental Health | |  | |  | |  | |  | |  | |  | |  |  | |  | |  | |
| Ever diagnosed with: | |  | |  | |  | |  | |  | |  | |  |  | |  | |  | |
| Depression | | 2.65 (1.62, 4.61) | | **<0.001** | | **2.79 (1.53, 5.10)** | | **0.001** | | 2.06 (0.88, 4.78) | | 0.094 | | **2.40 (1.17, 4.93)** | **0.017** | | **2.91 (1.54, 5.49)** | | **0.001** | |
| Anxiety | | 3.05 (1.86, 5.02) | | **<0.001** | | **3.58 (1.79, 7.16)** | | **<0.001** | | 2.04 (0.99, 4.20) | | 0.052 | | **2.59 (1.32, 5.06)** | **0.006** | | **3.71 (1.85, 7.42)** | | **<0.001** | |
| PTSD | | 1.49 (0.66, 3.36) | | 0.335 | | 1.55 (0.61, 3.94) | | 0.355 | | 1.34 (0.25, 7.23) | | 0.737 | | 0.80 (0.28, 2.34) | 0.688 | | 2.66 (0.81, 8.73) | | 0.108 | |
| Any mental health condition of interest | | **2.04 (1.38, 3.03)** | | **<0.001** | | **2.15 (1.31, 3.51)** | | **0.002** | | 1.55 (0.81, 3.00) | | 0.189 | | 1.60 (0.93, 2.74) | 0.091 | | **2.54 (1.48, 4.36)** | | **<0.001** | |
|  | |  | |  | |  | |  | |  | |  | |  |  | |  | |  | |
| Stress | |  | |  | |  | |  | |  | |  | |  |  | |  | |  | |
| At least one stressful event | | **2.24 (1.63, 3.07)** | | **<0.001** | | **2.15 (1.49, 3.10)** | | **<0.001** | | **2.32 (1.22, 4.43)** | | **0.011** | | **2.32 (1.44, 3.73)** | **<0.001** | | **2.15 (1.41, 3.27)** | | **<0.001** | |
| Total number of stressful events | | **1.11 (1.06, 1.17)** | | **<0.001** | | **1.13 (1.06, 1.20)** | | **<0.001** | | 1.05 (0.96, 1.15) | | 0.260 | | **1.13 (1.04, 1.24)** | **0.006** | | **1.09 (1.03, 1.16)** | | **0.002** | |
|  | |  | |  | |  | |  | |  | |  | |  |  | |  | |  | |
| Have had a combat deployment/ experienced combat | | 0.81 (0.53, 1.23) | | 0.350 | | 0.85 (0.52, 1.37) | | 0.506 | | 0.80 (0.32, 2.00) | | 0.639 | | 0.57 (0.24, 1.35) | 0.200 | | 0.99 (0.61, 1.61) | | 0.976 | |
| *p<0.05 considered significant; significant odds ratios are in bold | | | | | | | | | | | | | | | | | | | | |
