# Supplementary material for: Risk factors for positive depression screening across a shipboard deployment cycle
Source: BJPsych Open. 2019 Sep 20;5(5):e84. doi: 10.1192/bjo.2019.70 (PMC6788222; doi:10.1192/bjo.2019.70)
Supplement: Supplementary file 1 [file S205647241900070Xsup001.zip › S205647241900070Xsup001/Supp Table 2.docx]

| Supplementary Table 2. Longitudinal model describing risk factors for screening positive for depression among female respondents | | |
| --- | --- | --- |
|  | OR (95% CI) | p-value |
| Age groups |  |  |
| 17-20 | 4.46 (0.96, 20.71) | 0.056 |
| **21-22** | **11.98 (2.91, 49.36)** | **0.001** |
| **23-24** | **8.18 (2.13, 31.35)** | **0.002** |
| 25-30 | 3.02 (0.96, 9.53) | 0.059 |
| 31+ (referent) | N/A | N/A |
| Relationship status |  |  |
| In a relationship | 1.06 (0.50, 2.23) | 0.883 |
| Race |  |  |
| White (referent) | N/A | N/A |
| **Black** | **2.78 (1.22, 6.30)** | **0.015** |
| Hispanic | 2.90 (0.99, 8.53) | 0.053 |
| Other | 0.72 (0.31, 1.65) | 0.437 |
| Education |  |  |
| College graduate or higher | 1.17 (0.28, 4.85) | 0.832 |
| Rank |  |  |
| Enlisted (referent) |  |  |
| W1-W5, O1-O9 | 1.59 (0.30, 8.41) | 0.588 |
| Military experience |  |  |
| No deployments (referent) | N/A | N/A |
| **1 deployment** | **3.24 (1.33, 7.88)** | **0.001** |
| **2 or more deployments** | **4.56 (1.77, 11.77)** | **0.002** |
| Alcohol |  |  |
| Positive CAGE screening | 0.85 (0.28, 2.57) | 0.779 |
|  |  |  |
| Lighter than Moderate-heavy drinker (referent) | N/A | N/A |
| Moderate-heavy to heavy drinker | 1.54 (0.82, 2.87) | 0.178 |
|  |  |  |
| Have ever passed out/blacked out from drinking | 0.94 (0.47, 1.91) | 0.872 |
|  |  |  |
| Have consumed alcohol in the past year | 2.03 (0.67, 6.00) | 0.200 |
| Mental Health |  |  |
| Diagnosed at any previous point |  |  |
| **Diagnosed with an anxiety disorder** | **2.72 (1.09, 6.80)** | **0.032** |
| Stress |  |  |
| **At least one stressful event** | **3.59 (1.54, 8.34)** | **0.003** |
| Drug use |  |  |
| **Have ever used any drugs** | **0.20 (0.07, 0.52)** | **0.001** |
