# Supplementary material for: Risk factors for positive depression screening across a shipboard deployment cycle
Source: BJPsych Open. 2019 Sep 20;5(5):e84. doi: 10.1192/bjo.2019.70 (PMC6788222; doi:10.1192/bjo.2019.70)
Supplement: Supplementary file 1 [file S205647241900070Xsup001.zip › S205647241900070Xsup001/Supp Table 3.docx]

| Supplementary Table 3. Longitudinal model describing risk factors for screening positive for depression among male respondents | | |
| --- | --- | --- |
|  | OR (95% CI) | p-value |
| Age groups |  |  |
| 17-20 | 1.41 (0.40, 4.95) | 0.593 |
| 21-22 | 0.78 (0.30, 2.01) | 0.605 |
| 23-24 | 1.19 (0.52, 2.73) | 0.684 |
| 25-30 | 0.91 (0.47, 1.79) | 0.794 |
| 31+ (referent) | N/A | N/A |
| Marital status |  |  |
| Single, uncommitted (referent) | N/A | N/A |
| Single, committed relationship | 0.48 (0.21, 1.10) | 0.085 |
| **Single, living with partner** | **0.32 (0.13, 0.76)** | **0.010** |
| **Married** | **0.46 (0.24, 0.89)** | **0.022** |
| Divorced, separated, or widowed | 0.43 (0.12, 1.62) | 0.213 |
| Race |  |  |
| White (referent) | N/A | N/A |
| Black | 1.03 (0.48, 2.23) | 0.935 |
| Hispanic | 2.15 (0.96, 4.83) | 0.064 |
| Other | 2.04 (1.07, 3.88) | 0.030 |
| Longest amount of time away from partner (T1 and T3 only) |  |  |
| 1 month or less (referent) | N/A | N/A |
| Greater than 1 month | 1.23 (0.70, 2.26) | 0.434 |
| Education |  |  |
| High school or less | 0.82 (0.49, 1.39) | 0.461 |
| Some college, graduated from vocational school (referent) | N/A | N/A |
| College graduate or higher | 0.73 (0.35, 1.52) | 0.404 |
| Rank |  |  |
| Enlisted (referent) | N/A | N/A |
| W1-W5, O1-O9 | 0.67 (0.25, 1.76) | 0.416 |
| Military experience |  |  |
| No deployments (referent) | N/A | N/A |
| 1 deployment | 0.65 (0.30, 1.40) | 0.272 |
| 2 or more deployments | 0.48 (0.20, 1.13) | 0.092 |
| Alcohol |  |  |
| Positive CAGE screening | **2.79 (1.49, 5.24)** | **0.001** |
|  |  |  |
| AUDIT-C score ≥5 | 1.37 (0.87, 2.15) | 0.173 |
|  |  |  |
| Have ever passed out/blacked out from drinking | 0.61 (0.36, 1.01) | 0.056 |
|  |  |  |
| **Have consumed alcohol in the past year** | **2.42 (1.06, 5.55)** | **0.036** |
| Mental Health |  |  |
| **Any mental health condition of interest** | **3.11 (1.43, 6.80** | **0.004** |
| Stressful experience |  |  |
| At least one stressful event | 0.93 (0.51, 1.69) | 0.805 |
| **Total number of stressful events** | **1.18 (1.08, 1.29)** | **<0.001** |
|  |  |  |
| Drug use |  |  |
| Have ever used any drugs | 0.82 (0.38, 1.80) | 0.82 |
