# Supplementary material for: Risk factors for positive depression screening across a shipboard deployment cycle
Source: BJPsych Open. 2019 Sep 20;5(5):e84. doi: 10.1192/bjo.2019.70 (PMC6788222; doi:10.1192/bjo.2019.70)
Supplement: Supplementary file 1 [file S205647241900070Xsup001.zip › S205647241900070Xsup001/Supp Table 4.docx]

| Supplementary Table 4. Longitudinal model describing risk factors for screening positive for depression among respondents 24 years and younger | | |
| --- | --- | --- |
|  | OR (95% CI) | p-value |
| **Sex (female)** | **3.17 (1.65, 6.09)** | **<0.001** |
| Relationship status |  |  |
| In a relationship | 0.80 (0.44, 1.47) | 0.475 |
| Race |  |  |
| White (referent) | N/A | N/A |
| Black | 0.73 (0.32, 1.67) | 0.452 |
| Hispanic | 2.10 (0.90, 4.92) | 0.087 |
| Other | 2.78 (1.01, 7.68) | 0.048 |
| Longest amount of time away from partner (T1 and T3 only) |  |  |
| 1 month or less (referent) | N/A | N/A |
| Greater than 1 month | 1.45 (0.67, 3.13) | 0.340 |
| Military experience |  |  |
| No deployments (referent) | N/A | N/A |
| 1 deployment | 1.19 (0.60, 2.37) | 0.615 |
| 2 or more deployments | 0.82 (0.33, 2.04) | 0.669 |
| Alcohol |  |  |
| Positive CAGE screening | 1.56 (0.59, 4.12) | 0.3735 |
|  |  |  |
| Lighter than Moderate-heavy drinker (referent) | N/A | N/A |
| **Moderate-heavy to heavy drinker** | **1.71 (1.00, 2.90)** | **0.0485** |
|  |  |  |
| Have ever passed out/blacked out from drinking | 1.51 (0.80, 2.85) | 0.198 |
| Mental Health |  |  |
| Diagnosed with depression | 3.34 (0.92, 12.21) | 0.068 |
| Stress |  |  |
| **At least one stressful event** | **2.36 (1.28, 4.38)** | **0.006** |
| Drug use |  |  |
| Have ever used any drugs | 0.42 (0.16, 1.13) | 0.085 |
