# Supplementary material for: Risk factors for positive depression screening across a shipboard deployment cycle
Source: BJPsych Open. 2019 Sep 20;5(5):e84. doi: 10.1192/bjo.2019.70 (PMC6788222; doi:10.1192/bjo.2019.70)
Supplement: Supplementary file 1 [file S205647241900070Xsup001.zip › S205647241900070Xsup001/Supp Table 5.docx]

| Supplementary Table 5. Longitudinal model describing risk factors for screening positive for depression among respondents 25 years and older | | |
| --- | --- | --- |
|  | OR (95% CI) | p-value |
| Age | 0.95 (0.90, 1.00) | 0.054 |
| Sex (female) | 0.75 (0.37, 1.55) | 0.442 |
| Marital status |  |  |
| Single, uncommitted (referent) | N/A | N/A |
| Single, committed relationship | 0.57 (0.17, 1.87) | 0.354 |
| **Single, living with partner** | **0.29 (0.09, 0.97)** | **0.044** |
| **Married** | **0.35 (0.14, 0.86)** | **0.022** |
| **Divorced, separated, or widowed** | **0.22 (0.06, 0.83)** | **0.025** |
| Race |  |  |
| White (referent) | N/A | N/A |
| Black | 1.58 (0.66, 3.74) | 0.303 |
| **Hispanic** | **2.52 (1.02, 6.24)** | **0.046** |
| Other | 1.52 (0.76, 3.03) | 0.236 |
| Longest amount of time away from partner (T1 and T3 only) |  |  |
| 1 month or less (referent) | N/A | N/A |
| Greater than 1 month | 1.01 (0.51, 2.00) | 0.972 |
| Education |  |  |
| High school or less | 0.53 (0.26, 1.09) | 0.086 |
| Some college, graduated from vocational school (referent) | N/A | N/A |
| College graduate or higher | 0.94 (0.46, 1.93) | 0.860 |
| Rank |  |  |
| Enlisted (referent) | N/A | N/A |
| W1-W5, O1-O9 | 0.84 (0.33, 2.12) | 0.707 |
| Military experience |  |  |
| No deployments (referent) | N/A | N/A |
| 1 deployment | 1.57 (0.46, 5.39) | 0.475 |
| 2 or more deployments | 1.42 (0.42, 4.80) | 0.577 |
| Alcohol |  |  |
| Positive CAGE screening | 2.19 (0.94, 5.13) | 0.071 |
|  |  |  |
| Lighter than Moderate-heavy drinker (referent) | N/A | N/A |
| Moderate-heavy to heavy drinker | 1.14 (0.64, 1.99) | 2.05 |
|  |  |  |
| AUDIT-C score: ≥4 women, ≥5 men | 1.36 (0.70, 2.61) | 0.362 |
|  |  |  |
| Have ever passed out/blacked out from drinking | 0.52 (0.28, 0.97) | 0.040 |
|  |  |  |
| Have consumed alcohol in the past year | 1.50 (0.55, 4.11) | 0.4326 |
| Mental Health |  |  |
| **Any mental health condition of interest** | **3.86 (1.67, 8.91)** | **0.002** |
| Stressful experience |  |  |
| At least one stressful event | 0.85 (0.39, 1.85) | 0.674 |
| **Total number of stressful events** | **1.20 (1.08, 1.33)** | **<0.001** |
|  |  |  |
| Have had a combat deployment/experienced combat | 1.35 (0.64, 2.82) | 0.428 |
| Drug use |  |  |
| Have ever used any drugs | 0.91 (0.29, 2.84) | 0.873 |
